# Structural and electrochemical investigation of benzimidazole picolinic acid derivatives for sustainable energy storage applications

**DOI:** 10.1038/s41598-025-01503-3

**Published:** 2025-07-01

**Authors:** P. Divya, A. Amala Jeya Ranchani, Hijaz Ahmad, V. S. Jeba Reeda, Taha Radwan, Divya Haridas

**Affiliations:** 1 Department of Physics, Saveetha School of Engineering, Saveetha Institute of Medical and Technical Sciences (SIMATS), Thandalam, Chennai India; 2 Near East University, Operational Research Center in Healthcare, Near East Boulevard, 99138 Nicosia/Mersin 10, Turkey; 3 Department of Physics, Easwari Engineering College, Ramapuram, Chennai, 600 089 Tamil Nadu India; 4 Department of Management Information Systems, College of Business and Economics, Qassim University, 51452 Buraydah, Saudi Arabia

**Keywords:** Cyclic voltametric analysis, Photoluminescence, Nyquist plot, DFT, Chemistry, Materials science, Physics

## Abstract

Supercapacitors are widely employed in electric vehicles and portable electronics due to their rapid charge–discharge capability, high power density, and energy efficiency; however, many still depend on non-renewable materials. This study reports the synthesis and comprehensive characterization of benzimidazole picolinic acid (BPEP) as a sustainable, high-performance electrode material for supercapacitors. Optical and electrical analyses reveal significant π → π* and n → π* transitions, with prominent UV–visible absorption peaks at 282 nm (experimental) and 294 nm (theoretical). BPEP exhibits excellent light absorption, achieving 99% light-harvesting efficiency at 300 nm and an extinction coefficient of 2250, underscoring its strong optical potential. Frontier Molecular Orbital (FMO) analysis indicates a band gap of 3.9 eV, reflecting notable chemical hardness and thermal stability. Additionally, the negative chemical potential (μ = –4.377 eV) and high electrophilicity index (ω = 5.249 eV) suggest enhanced chemical reactivity and stability that key attributes for energy storage. Electrochemical impedance analysis reveals a maximum specific capacitance of 125.45 F/g at a scan rate of 10 mV/s, confirming efficient charge storage behavior. Vibrational analysis and optimized geometry further affirm the structural integrity of BPEP. Collectively, these results highlight the optical, topological, and electrochemical merits of BPEP, establishing it as a promising eco-friendly candidate for next-generation supercapacitor electrodes.

## Introduction

Because of population growth and severe environmental pollution driven by the increasing need for efficient energy storage materials, our research team is advancing studies in advanced materials for supercapacitance applications^1^. Many researchers are investigating state of the art technologies to accelerate energy conservation and improve energy efficiency, ultimately elevating the standard of living^2^. To address the issue of sustainable energy, it is necessary and beneficial to explore clean, green, and renewable resources. However, the output and efficiency of these energy sources are highly dependent on geographic factors^3–5^. Electrochromic (EC) devices play a crucial role in modern technology by enabling active control of sunlight glazing, leading to energy savings^6^.Supercapacitors are divided into pseudocapacitors^7^, electrochemical double^8^ layer capacitors (EDLCs), and^9^ hybrid capacitors according to the energy storage mechanism. Electrode^10,11^ materials for supercapacitors (SCs) are unique to each storage method include carbon nanotubes, graphene. Their unique feature lies in their ability to achieve multiple colors through voltage adjustments during operation. Recently, researchers have focused on integrating electrochemical energy storage capabilities into EC devices, thereby enhancing the multifunctionality of these devices. Effective charge storage is made possible by EC devices’ reversible redox reactions at the electrode surface, which provide pseudocapacitance during optical modulation. EC devices generate pseudocapacitance during optical modulation through a reversible redox reaction at the electrode surface, allowing efficient charge storage. Scientific study has focused a lot of interest on organic–inorganic hybrid materials because of their amazing structural plasticity and electrochemical characteristics. Benzimidazole derivatives and picolinic acid, with their impressive electroactive sites and robust structures, hold promise for supercapacitance applications. Functionalization and composite formation can further enhance their performance.

Despite the promising performance of BPEP, developing such materials presents challenges including achieving consistent porosity, controlling crystal morphology, and optimizing redox-active sites. Compared to existing carbon-based or metal oxide supercapacitor materials, organic–inorganic hybrids like BPEP require precise synthesis conditions to ensure high conductivity and mechanical stability. Additionally, scalability and cost-effectiveness remain significant hurdles in transitioning from laboratory-scale success to commercial application.

The primary objective of this study is to design and evaluate a novel benzimidazole-picolinic acid–ethanol (BPEP) composite, with a focus on understanding its structural, optical, and electrochemical properties for energy storage applications. The study also investigates the composite’s stability, capacitance behavior, and charge storage performance compared to existing supercapacitor materials.

## Experimental section details

### BPEP crystals growth

Analytical grade precursors, benzimidazole and picolinic acid were combined in an equimolar ratio to create BPEP. First, 0.9003 g of benzimidazole was added after 0.9312 g of picolinic acid had been dissolved in ethanol. To guarantee complete mixing, the growth fluid was continuously swirled for six hours before being filtered to remove impurities. When the solvent had evaporated entirely and the BPEP salt had dried at room temperature, a white, crystalline salt was the result. Multiple recrystallization procedures were required to attain the desired purity of the produced crystal. Solution remained saturated at room temperature also allowed to slowly evaporate in order to promote bulk crystal formation. A superior BPEP crystal was formed after 30 days and then it is powdered.

### Specifics of the experiment

Crystalline nature of the crystal is determined by using XRD model BRUKER-binary V4 (.RAW). Perkin Elmer FT-IR spectrometer and the risk-averse KBr pellet approach, the FT-IR emission spectra of BPEP were investigated spanning the wavelength along 4000–500 cm⁻^1^. Additionally, the FT-Raman spectrum, which uses an air-cooled Nd:YAG laser operating at roughly 1064 nm and has a wavelength spectrum spanning from 4000 to 50 cm⁻^1^, was separately recorded using a Bruker RFS-27. Using an UV–vis spectrophotometer, UV spectra covering the wavelength range of 200–800 nm were obtained. EIS is a useful tool for probing the electrical properties of materials. The 0.009-cm-thick sample, which has been sliced into 1-cm-squares, is positioned between two stainless steel electrodes. Using a 1.0 V AC signal to measure impedance between 42 Hz and 1 MHz can help determine the films’ bulk ionic conductivity (σ) and dielectric characteristics (ɛ). The computations were carried for PL using Photoluminescence Spectrometer (PL) Model LS45.

## Theoretical details

With Gaussian ‘09 software, DFT computations were carried out at B3LYP level on the basis set 6–311 + + G(d,p)^12^. GaussView 5.0 software was used to evaluate PES studies^13^. Using VEDA4^14^ vibrational spectrum peak assignments were completed effectively. Charge transfer (CT) interactions such as LP → BD* and BD → BD* (where LP represents lone pair orbitals, BD represents bonding orbitals, and BD* represents antibonding orbitals) were identified by NBO analysis utilizing the NBO 3.1 interface^15^. Frontier molecular orbital (FMO) theory study was performed also isosurface maps were generated using the VMD program, while the Multiwfn tool, known for its versatility in wave function analysis, was employed to examine the LOL, RDG, ELF^16,17^.

## Results and discussion

### Structural conformation studies

#### Powder XRD studies

The XRD pattern of BPEP is depicted in the image Figure S1. The Table 1 comprehensively analyzes powder X-ray diffraction (XRD) data for a material. It includes the position in degrees 2-theta (°2 Th.), the corresponding peak heights in counts (cts), the FWHM on left side of the peak in degrees 2-theta, the d-spacing in angstroms (Å), and the relative intensity (%) of each peak. The data is essential for describing the material’s crystalline structure, which directly influences its electrochemical properties. Well-defined crystalline structures and precise d-spacing values in materials used in supercapacitors can improve the ability to transport and store ions. The relative intensities provide information on the prevailing crystallographic planes, which can have an impact on the material’s surface area and porosity. Efficient charge storage and rapid charge–discharge cycles in supercapacitors require a high surface area and optimum porosity. Peaks with high relative intensities, such as the one at 21.7206° 2 theta with a relative intensity of 100%, indicate the presence of dominant phases that could have a major impact on the material’s capacitance performance.

**Table 1:** Interplanar spacing (d hkl) from XRD, percentage of variation of d, and FWHM.

Pos. [2 Th.]	Height [cts]	FWHM left [2 Th.]	d-spacing [Å]	Rel. int. [%]
11.2844	1442.99	0.1181	7.84145	40.54
12.2731	761.51	0.1574	7.21185	21.39
13.3759	191.11	0.3936	6.61964	5.37
16.8467	109.57	0.4723	5.26287	3.08
18.6940	278.62	0.1181	4.74677	7.83
18.8809	257.70	0.1181	4.70019	7.24
20.1415	502.17	0.1181	4.40877	14.11
20.8965	147.56	0.3149	4.25115	4.15
21.7206	3559.66	0.1181	4.09170	100.00
22.3361	244.57	0.1968	3.98032	6.87
22.6942	391.45	0.1181	3.91831	11.00
24.3403	542.60	0.1181	3.65693	15.24
24.7274	1492.40	0.1181	3.60054	41.93
26.9103	363.14	0.1574	3.31324	10.20
27.2404	358.95	0.1181	3.27383	10.08
27.8789	836.54	0.2362	3.20029	23.50
28.1800	452.79	0.1181	3.16677	12.72
30.2104	364.56	0.3542	2.95841	10.24
32.1702	192.32	0.1181	2.78251	5.40
33.5381	170.71	0.1181	2.67209	4.80
34.9994	216.36	0.1968	2.56380	6.08
36.1404	139.23	0.3149	2.48544	3.91
38.4194	163.87	0.1574	2.34309	4.60
40.1618	93.05	0.2362	2.24536	2.61
41.2825	163.24	0.1574	2.18696	4.59
42.2928	189.58	0.1574	2.13703	5.33
43.7594	130.80	0.1574	2.06874	3.67
47.2454	56.40	0.2362	1.92392	1.58
50.5860	115.97	0.3149	1.80443	3.26
53.7283	1217.62	0.1574	1.70608	34.21

#### Optimized geometry

**Fig. 1:**
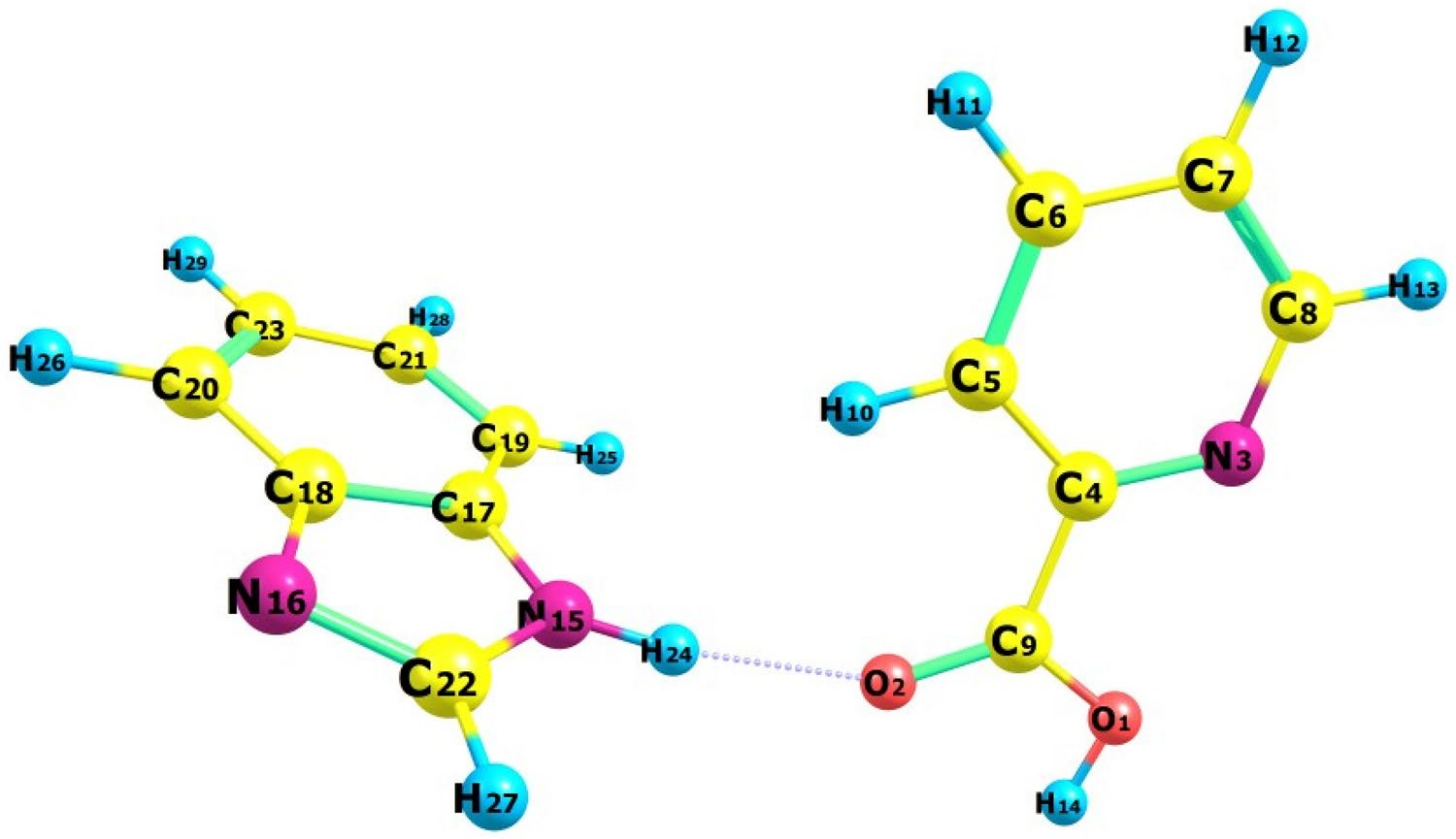
Optimized structure of BPEP.

Considerable insights are revealed by optimizing the geometric characteristics of the BPEP molecule through the use of DFT. Table 2 provides information on the bond lengths of BPEP molecules also bond angles are displayed in Table 2 respectively. The atomic numbering scheme for BPEP, which is based on DFT calculations, appears in Fig. 1. The variation in C–C bond lengths in picoline acid is caused by the ring’s adherence to electronegative N & O atoms. It is noteworthy that compared to other C–C bonds, the C_4_–C_9_ bond length (1.4988 Å) is longer. The C_17_–C_18_ bond length (1.4154 Å) in the benzimidazole ring is likewise greater than that associated with other C–C bonds. Specifically, the bond angles C_4_–N_3_–C_8_ (117.6°) and C_4_–C_5_–C_6_ (118.2°) are lower than the expected ideal, suggesting slight angular strain or electronic perturbations introduced by the presence of nearby electronegative atoms (N and O)^18^.

**Table 2:** Optimized bond lengths (Å) and bond angle (°) by B3LYP/6–311 + + g(d,p) of BPEP.

Bond length	B3LYP/6-311G + + (d,p) Theo	Bond angle	B3LYP/6-311G + + (d,p) Theo
O_1_-C_9_	1.336	C_9_-O_1_-H_14_	106.9744
O_1_-H_14_	0.969	C_9_-O_2_-H_24_	148.9259
O_2_-C_9_	1.217	C_4_-N_3_-C_8_	117.6
O_2_-H_24_	1.962	N_3_-C_4_-C_5_	123.6
N_3_-C_4_	1.3378	N_3_-C_4_-C_9_	117.8036
N_3_-C_8_	1.3313	C_5_-C_4_-C_9_	118.6458
C_4_-C_5_	1.3969	C_4_-C_5_-C_6_	118.2
C_4_-C_9_	1.4988	C_4_-C_5_-H_10_	120.432
C_5_-C_6_	1.3914	C_6_-C_5_-H_10_	121.3375
C_5_-H_10_	1.0816	C_5_-C_6_-C_7_	118.6713
C_6_-C_7_	1.3904	C_5_-C_6_-H_11_	120.3769
C_6_-H_11_	1.0836	C_7_-C_6_-H_11_	120.9518
C_7_-C_8_	1.3969	C_6_-C_7_-C_8_	118.5854
C_7_-H_12_	1.0835	C_6_-C_7_-H_12_	121.2574
C_8_-H_13_	1.086	C_8_-C_7_-H_12_	120.1572
N_15_-C_17_	1.383	N_3_-C_8_-C_7_	123.4
N_15_-C_22_	1.373	N_3_-C_8_-H_13_	116.1119
N_15_-H_24_	1.015	C_7_-C_8_-H_13_	120.5112
N_16_-C_18_	1.388	O_1_-C_9_-O_2_	122.1746
N_16_-C_22_	1.308	O_1_-C_9_-C_4_	114.0309
C_17_-C_18_	1.4154	O_2_-C_9_-C_4_	123.7944
C_17_-C_19_	1.396	C_17_-N_15_-C_22_	106.4868
C_18_-C_20_	1.3992	C_17_-N_15_-H_24_	127.1001
C_19_-C_21_	1.3904	C_22_-N_15_-H_24_	126.3782
C_19_-H_25_	1.084	C_18_-N_16_-C_22_	104.6922
C_20_-C_23_	1.3889	N_15_-C_17_-C_18_	104.8111
C_20_-H_26_	1.0835	N_15_-C_17_-C_19_	132.8
C_21_-C_23_	1.4088	C_18_-C_17_-C_19_	122.3531
C_21_-H_28_	1.0842	N_16_-C_18_-C_17_	110.1424
C_22_-H_27_	1.0809	N_16_-C_18_-C_20_	130.1
C_23_-H_29_	1.0841	C_17_-C_18_-C_20_	119.8508
		C_17_-C_19_-C_21_	116.826
		C_17_-C_19_-H_25_	121.864
		C_21_-C_19_-H_25_	121.3085
		C_18_-C_20_-C_23_	118.0618
		C_18_-C_20_-H_26_	120.2754
		C_23_-C_20_-H_26_	121.6628
		C_19_-C_21_-C_23_	121.5098
		C_19_-C_21_-H_28_	119.2694
		C_23_-C_21_-H_28_	119.2206
		N_15_-C_22_-N_16_	113.8674
		N_15_-C_22_-H_27_	121.1807
		N_16_-C_22_-H_27_	124.9519
		C_20_-C_23_-C_21_	121.3984
		C_20-_C_23_-H_29_	119.5757
		C_21_-C_23_-H_29_	119.0256

In this work, the computed C = O double bond length (O_2_-C_9_) is 1.217 Å, a little bit lower than compared to the C-O single bond length (O_1_-C_9_) of 1.336 Å. This difference occurs because, in the case of a C–O single bond, the oxygen atom takes electrons away from several carbon atoms^19^. This causes the force constant to fall, which raises the bond length. In contrast, only one carbon atom is stripped of its electrons by the oxygen atom in a C = O bond, which reduces the bond’s length by raising the force constant. These results imply that double bonds are more robust and smaller than single bonds. The NH bond length (N_15_-H_24_ = 1.015 Å) is somewhat longer than the OH bond (O_1_-H_14_ = 0.969 Å), but the OH bond remains more polar owing to the larger electronegativity distinction between O & H. The C_5_-H_10_ link is the smallest (1.0816 Å) in the picoline acid ring due to intramolecular C_5_-H_10_…O_2_ H-bond couplings.

Considerably shorter than the usual vdW separation (3 Å), the O_2_‧‧‧H_24_-N_15_ distance (1.962 Å) encourages intermolecular H-bonding interactions, which are essential for creating molecules with increased reactivity^20^. Because of the rearrangement of certain charges on the nitrogen atom when the unpaired electron obtains greatly delocalized, protonation minimizes the N_15_-C_17_ (1.383 Å), N_15_-C_22_ (1.373 Å), N_16_-C_18_ (1.388 Å), & N_16_-C_22_ (1.308 Å) bonds relative to the typical C-N bond length (1.47 Å). The heterocyclic ring’s bond angles, N_3_-C_4_-C_5_ (123.6°), N_3_-C_8_-C_7_ (123.4°), N_16_-C_18_-C_20_ (130.1°), and N_15_-C_17_-C_19_ (132.8°), are all slightly deformed and not exactly hexagonal despite the N atom’s existence^21^.

#### Vibrational analysis

Vibrational spectroscopy is a common technique in organic compound identification, kinetics analysis, and molecular conformation determination. In our investigation, the 29-atom compound BPEP exhibits 81 vibrational modes along with C1 point group symmetry. A stable chemical structure is suggested by the C1 symmetry. The vibrational study of the BPEP compound was carried out using DFT technique as reported in Table 3. Figure S2 and S3 displays the BPEP calculated also observed spectra. The predicted frequencies were found to be somewhat comparable to the frequencies noticed during the experiment when a contrast of the calculated and experimental values took place for the strongest peaks.

**Table 3:** Observed and calculated vibrational frequencies of BPEP by B3LYP/6–311 + + G(d,p) basis set.

	Experimental		B3LYP/ 6–311 + g(d,p)		PED(%)^b^
Mode	IR	Raman	Unscaled	Scaled^a^	
81	3487 (w)		3641	3495	νOH (100)
80	3279 (s)		3521	3380	νNH (99)
79	3149 (w)		3260	3130	νCH (99)
78			3221	3092	νCH (77) + νCH (17)
77			3208	3080	νCH (16) + νCH (16) + νCH (54) + νCH (14)
76	3072 (w)		3204	3076	νCH (71) + νCH (19)
75		3069 (s)	3193	3065	νCH (56) + νCH (32)
74			3191	3063	νCH (37) + νCH (17) + νCH (39)
73			3182	3055	νCH (11) + νCH (36) + νCH (52)
72			3177	3050	νCH (43) + νCH (45)
71	2930 (s)		3163	3036	νCH (17) + νCH (51) + νCH (30)
70	1629 (w)	1622 (w)	1665	1600	νCC (45) + νOC (19)
69	1605 (w)	1580 (w)	1660	1595	νOC (54)
68	1564 (s)		1625	1562	νCC (32) + βCCC(12) + βCNC(12)
67	1528 (w)		1614	1551	νCC (47) + βCCC (10) + βHCC(11) + βCNC(11)
66		1522 (w)	1604	1541	νNC (47) + νCC (23)
65		1495 (vw)	1548	1488	βHNC (27) + βHCC (19)
64	1457 (vw)	1453 (w)	1513	1454	βHNC (10) + βHCC (11) + βHCC (24) + βHCC (10)
63		1437 (vw)	1501	1442	νNC (16) + βHCC (17) + βHCC (11) + βHCN (26)
62			1480	1422	βHCC (13) + βHCC (28) + βHCN (22)
61		1417 (vw)	1479	1421	νNC (18) + βCNC (15) + βHCC (13) + βHCN (17)
60	1380 (vs)	1390 (w)	1452	1395	νNC (46) + νNC (15)
59			1417	1362	νOC (23) + νCC (13) + βOCC (11) + βHOC (21)
58		1347 (w)	1390	1336	νCC (23) + βCNC (10)
57		1284 (s)	1339	1287	νNC (34) + βHCC (11) + βHCN (19)
56			1331	1279	νNC (17) + βHCC (14) + βHCN (33)
55			1299	1248	βHNC (22)
54			1290	1240	νCC (12) + νCC (10) + νNC (11) + νNC (27)
53	1231 (s)		1280	1230	νCC (33) + βHCC (25) + βHCC (12)
52		1189 (w)	1221	1173	νCC (31) + βHOC (15) + βCNC (13)
51			1213	1166	νCC (12) + νNC (10) + βHCN (13)
50	1154 (vw)		1207	1160	νCC (11) + νCC (13) + βHCC (15) + βHCC (34) + βHCC (22)
49		1148 (w)	1192	1146	βHCC (18) + βHCC (26) + βHCC (11)
48		1099 (w)	1141	1097	νCC (15) + νCC (14) + βHCC (17) + βHCC (16)
47			1133	1089	νNC (10) + νCC (17) + νCC (10) + βHCC (16) + βHCC (18)
46			1129	1085	νNC (56) + βHNC (15) + βHCN (15)
45		1052 (w)	1103	1060	νOC (42) + βHOC (21)
44		1015 (s)	1062	1021	βCCC (52) + νCC (10)
43	999 (vs)	1000 (s)	1048	1007	τHCCC (26) + τHCCC (35) + τHCCN (18)
42			1029	989	βNCN (11) + βCCC (12) + βCCN (17) + βCNC (17)
41			1013	973	τHCCC (26) + τHCCN (19) + τHCNC (31)
40			1011	972	τHCCC (15) + τHCCC (14) + τHCCC (26) + τCCCC (20) + τCCNC (11)
39			1005	966	νNC (11) + νCC (11) + βCCC (35) + βNCC (12) + βCCC (13)
38		931 (w)	976	938	τHCCC (20) + τHCCC (27) + τHCCC (32)
37	922 (vw)		955	918	νNC (10) + βCNC (14) + βNCN (52)
36	899 (vw)		951	914	τHCCC (29) + τHCCC (14) + τHCCN (10) + τHCNC (25) + τCNCC (15)
35	868 (w)		902	867	νNC (12) + βCCC (30) + βCCC (32)
34	845 (vw)	846 (w)	893	858	τHCCC (31) + τHCCC (20) + τHCCC (21)
33			863	829	τHNCN (24) + τHCNC (51) + τCNCN (15)
32			854	821	τHCCC (15) + τHCCN (14) + τHCNC (12) + τOOCC (25) + τCCNC (18)
31			847	814	τHNCN (70) + τHCNC (23)
30	780 (vw)	778 (s)	797	766	νCC (18) + βCCC (10) + βNCC (10) + βCCC (31)
29			793	762	τHCCC (14) + τCCCC (17) + τCNCN (31) + τCNCC (15)
28			791	760	νCC (14) + νCC (16) + βCNC (17) + βCNC (18)
27		746 (vw)	780	750	τHCCC (11) + τHCCC (30) + τHCCC (25) + τCNCC (18)
26	744 (s)		769	739	τHCCC (12) + τHCCC (10) + τHCCN (31) + τCCCC (10) + τCNCC (17) + τCCNC (10)
25	696 (s)		727	699	τHOCO (26) + τCCCC (10) + τCNCC (21) + τOOCC (22)
24			659	633	βOCC (16) + βNCC (19) + βCNC (10) + βOCC (19)
23			649	624	τHCNC (21) + τCNCN (17) + τCCNC (12) + τCOCN (16)
22		620 (w)	643	618	νCC (15) + νCC (16) + βCNC (14) + βCNC (13)
21			640	615	τHOCO (69)
20	613 (s)		639	614	βOCC (16) + βNCC (12) + βCCC (12)
19	554 (s)		600	577	τHCCC (12) + τCCCN (32) + τCCNC (19)
18	531 (vw)	545 (w)	561	539	νNC (15) + βCCC (18) + βCCC (20) + βCCC (10)
17	506 (vw)		505	485	βOCC (27) + βOCC (22) + βCCN (21)
16			448	431	τHCCC (13) + τCCCC (21) + τCNCC (12) + τOOCC (18) + τCCNC (20)
15		414 (w)	446	429	τHCCC (11) + τHCCC (11) + τCCCC (23) + τCNCC (42)
14			425	408	νNC (10) + βCCC (26) + βCCN (32)
13			419	403	τHCNC (17) + τNCCC (32) + τCCCC (19) + τCCNC (10)
12			389	374	νCC (32) + βCNC (24) + βOCC (19)
11		281 (vw)	270	259	τNCNO (26 + τCNCN (11) + τCCNC (13) + τCOCN (29)
10		240 (vw)	229	220	τCCCN (43) + τCCCC (12) + τCCNC (25)
9			219	210	βOCC (11) + βOCC (19) + βCCN (53)
8			159	153	τNCCC (27) + τCCCC (18) + τCCNC (30)
7		107 (s)	105	101	βOCC (30)
6		81 (vs)	77	74	βCNO (21) + τOCCC (47) + τNOCC (13)
5			61	59	βNOC (15) + τNCNO (45) + τCOCN (29)
4			35	34	βCNO (42) + τOCCC (40) + τNOCC (11)
3			16	15	βCNO (15) + τNOCC (62)
2			14	13	βNOC (54) + τCNOC (27)
1			5	5	βNOC (28) + τCNOC (49) + τNOCC (11)

^a^Scalingfactor:0.96 above 3000 cm^−1^ and 0.961 below 3000 cm^−1^ for wb97xd/6–311 + g(d,p), ^b^ν-Stretching, -bending, ω–out of plane, -torsion.

##### C = O and C-O vibration

Depending upon BPEP structure and conformation, ν(C = O) frequency is usually reported around 1800–1600 cm^−1^^22^ range. ν(C = O) vibration, which is estimated around 1595 cm^−1^ with a 54% PED, is represented by a faint band in the spectrum at 1629 cm^−1^(FT-IR) & 1580 cm^−1^(FT-Raman). H-bonding interactions between O_2_‧‧‧H_24_-N_15_ molecules are responsible for the minor variation observed in the experimental spectra. Furthermore, a weak band that shows up in the experimental data at 1052 cm^−1^ is predicted to be at 1060 cm^−1^ using a 42% PED. These modes are attributed to bending modes of the HOC picoline ring combined with ν(CO) vibrations.

##### CH vibration

Aromatic C-H stretching vibrations often seen around 3100–3000 cm^−1^^23^. Bands found at 3149 cm^−1^ (weak), 3072 cm^−1^ (weak), 2930 cm^−1^ & 3069 cm^−1^ (strong) in the FT-IR spectra are indicative of aromatic ν(C-H) vibrations. The estimated mode numbers (71–79) match ν(C–H) bands that have high PED. The existence of intramolecular C_5_-H_10_…O_2_ H-bond interactions accounts for the small discrepancies among measured and reported wavenumbers. The HCC bending area was detected at 1602–1028 cm^−1^^24^. β(H-C–C) vibrations at 1160 cm^−1^, 1146 cm^−1^, and 1097 cm^−1^ were found using the DFT approach. These were identified in the FT-IR spectra at 1154 cm^−1^ (very weak), also in FT-Raman spectrum around 1148 cm^−1^ (weak) and 1099 cm^−1^ (weak), each having excellent PED values.

##### NH and OH vibration

The FT-IR spectrum typically exhibits less significance for N–H bonding when in contrast to C-H or C–C bonding^25^. Strong wavenumber around 3500–3300 cm⁻^1^ range are usually observed in ν(N–H) vibrations. The FT-IR spectra show a prominent ν(N–H) band at 3279 cm⁻^1^. With a positive divergence of 101 cm⁻^1^ from the observed value and a redshift of 21 cm⁻^1^ from the predicted value, the equivalent theorized wavenumber for ν(N–H) is 3380 cm⁻^1^. Strong interactions between O_2_‧‧‧H_24_-N_15_ intermolecular H-bonds are the cause of this divergence. The molecule’s O–H (hydroxyl group) exhibits significant absorption in the 3550–3200 cm⁻^1^^26^range. The FT-IR spectrum revealed a faint band at 3487 cm⁻^1^ also PED computation indicates bands are pure.

##### C = N and C-N vibration

ν(C = N) vibration wavenumber are commonly found at about 1555 cm⁻^1^. v(C = N) vibration band in this work was determined to be weak at 1522 cm⁻^1^ in the FT-Raman spectrum and to have a 47% PED at 1541 cm⁻^1^ utilizing DFT calculations. Furthermore, the basic ν(C-N) vibration bands are often positioned between 1382 −1266 cm⁻^1^^27^. The computed ν(C-N) values for the BPEP compound at the DFT level are 1287 cm⁻^1^ and 1395 cm⁻^1^. Experiments revealed that these bands, which all displayed good PED values, were seen at 1380 cm⁻^1^ (vs) in the FT-IR spectrum, 1390 cm⁻^1^ (weak), and 1284 cm⁻^1^ (strong) in the FT-Raman spectrum.

##### C–C vibration

C = C vibrations are especially fascinating when double bonds are linked to a ring. C–C and C = C stretching modes can be observed between 1650–1200 cm⁻^1^^28^. It focuses on the structural changes of the ring rather than the kind of substituents that define the exact position of these modes. The FT-IR and Raman spectra display C–C stretching vibrations at 1629 cm⁻^1^ (weak), 1564 cm⁻^1^ (strong), 1528 cm⁻^1^ (weak), and 1231 cm⁻^1^ (strong), respectively. The FT-Raman spectrum displays these vibrations at 1622 cm⁻^1^ (weak), 1347 cm⁻^1^ (weak), and 1189 cm⁻^1^ (weak), with PED values of 45%, 32%, 47%, 33%, 23%, and 31%, respectively. These values perfectly match calculated wavenumbers at 1600, 1562, 1551, 1336, 1230, and 1173 cm⁻^1^. FT-Raman records the C–C-C ring in-plane bending as a substantial peak at 1015 cm⁻^1^ (strong), which is scaled at 1021 cm⁻^1^. Furthermore, in line with the literature, these frequencies show different ring replacements that impact vibrational ring modes^29,30^.

### Transistion studies

#### UV analysis

**Fig. 2:**
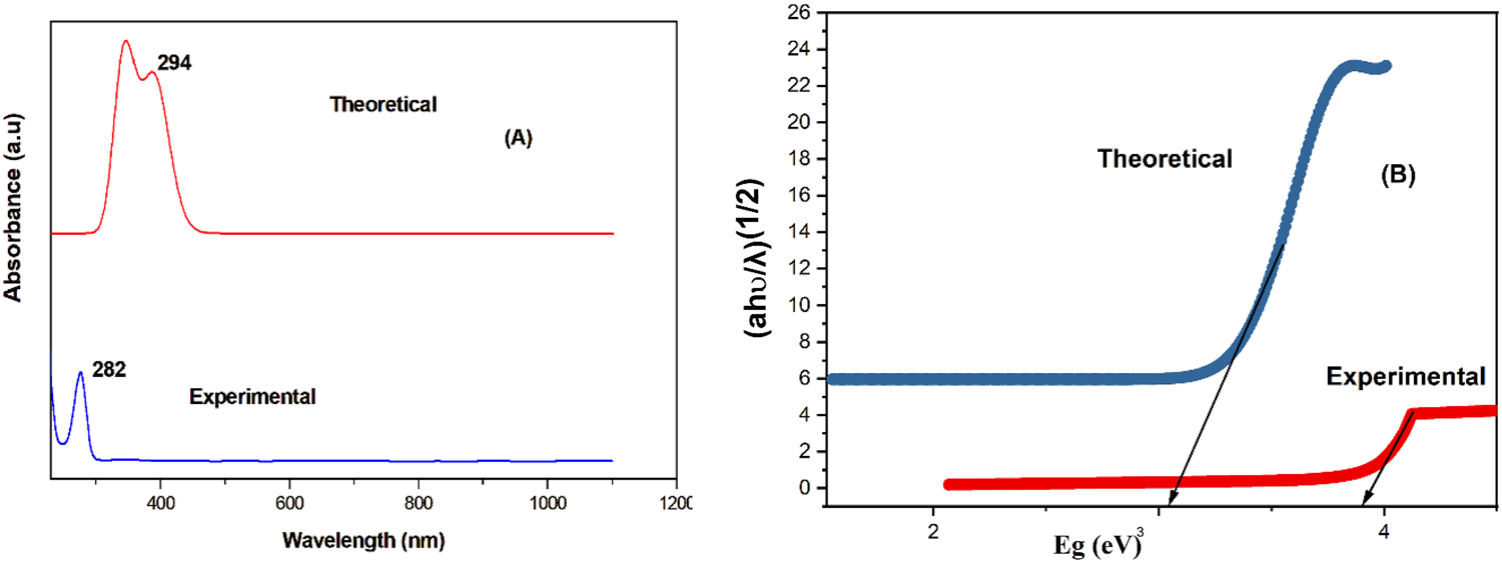
(**A**) Experimental and theoretical UV–visible spectrum of BPEP, (**B**) Experimental and theoretical tauc,s plot of BPEP.

UV–visible spectrum analysis is a critical tool for assessing protein integrity and how it interacts with ligand molecules since it is necessary for comprehending charge transfer and electronic excitation^31,32^. UV–visible spectroscopy, in particular, is commonly employed to analyze these relationships by monitoring modifications to the absorption parameters of drug-receptor complexes. The spectra in ethanol exhibited absorption bands at 282 nm. Substantial electronic changes were observed at 294 nm in the theoretical spectrum with an oscillator strength (f) of 0.0012 shown in Fig. 2 and Table 4. These transitions were mainly caused by the change from (H-2) to (LUMO) (99%). Further large transition occurred at 322 nm (f = 0.0005), primarily from (H-1) to LUMO (97%). It is evident from these transitions that the n → π* and π → π* transitions, which mainly involve carboxylic acid group also the benzimidazole ring where in intermolecular interactions happens through N_15_-H_24_…O_2_, combine to form the UV–Vis bands of BPEP. The intricate interactions inside the BPEP molecule are better understood because of this thorough examination of the electronic transitions. The calculated energy band gap was determined to be 3.9 eV, which is in close agreement with the 3.05 eV band gap that was previously noted.

**Table 4:** UV–vis excitation energy and oscillator strength of BPEP.

Experimental		Energy (cm ^−1^)	Theoretical		Oscillator strength f	Symmetry	Assignments
λ_max_ (nm)	Band gap (eV)		λ_max_ (nm)	Band gap (eV)			
		30,581	327		0.0005	Singlet-A	HOMO- > LUMO (97%)
		31,047	322		0.0005	Singlet-A	H-1- > LUMO (97%)
282	3.05	33,989	294	3.9	0.0012	Singlet-A	H-2- > LUMO (99%)

##### Extinction coefficient

**Fig. 3:**
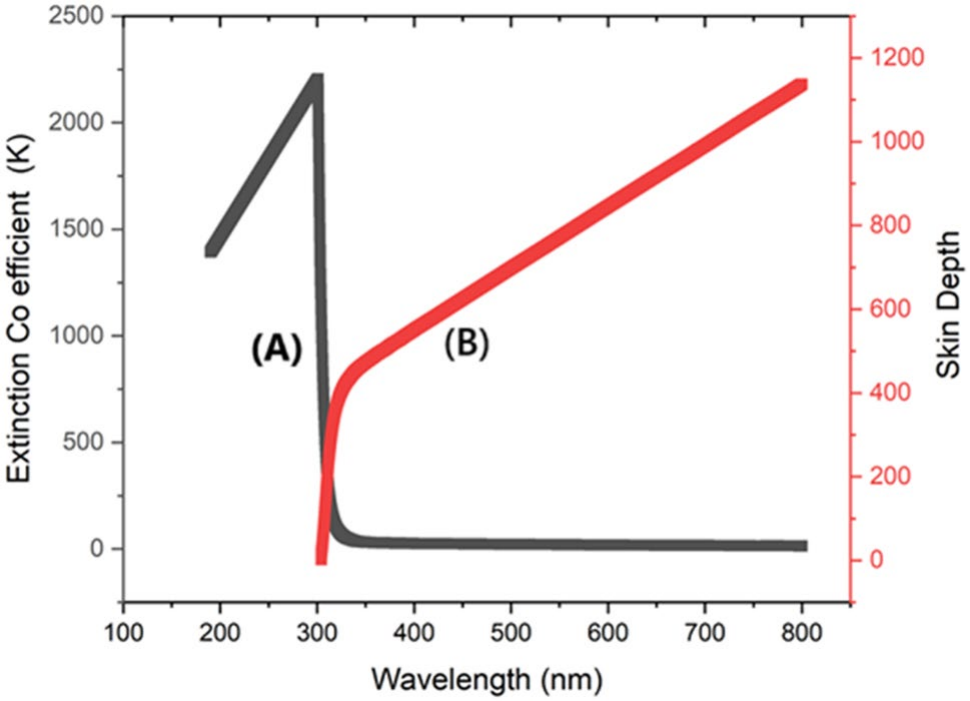
(**A**) Extinction coefficient of BPEP, (**B**) Electron Conduction Depth.

The extinction coefficient quantifies the degree to which a substance absorbs light at a specific wavelength and is directly correlated with the UV absorbance value which is dissipated in Fig. 3A. A high extinction coefficient signifies a significant capacity for light absorption, which corresponds to a high level of UV absorbance. The extinction coefficient reaches its peak value of 2250 at a wavelength of 300 nm, most likely as a result of strong electronic transitions or resonances occurring at this specific wavelength. When the wavelength exceeds 300 nm (Fig. 5A), these transitions become less efficient, resulting in a sharp fall of the extinction coefficient to zero. Materials having high attenuation coefficients at specific wavelengths can improve the efficiency of light-driven processes in supercapacitor applications. These processes include photothermal or photoelectrochemical reactions, and the materials increase them by enhancing light absorption and conversion inside the electrode materials. Enhanced energy storage capacity and overall performance of the supercapacitor can result from this.

##### Skin depth

Skin depth, commonly referred to as penetration depth, quantifies the extent to which light may permeate a substance prior to being absorbed which is shown in Fig. 3B. The UV absorbance value and skin depth have an inverse relationship: higher absorbance corresponds to a shallower skin depth. As the wavelength of light increases from 310 to 800 nm, the absorbance of various substances decreases, resulting in a progressive increase in the depth at which light may penetrate the skin. This depth ranges from around 310 nm to a maximum depth of 1150 nm at wavelength 800 nm (Fig. 5B). The greater skin depth observed at longer wavelengths allows for deeper light penetration into the substance. Materials having greater skin depth at specific wavelengths can improve the efficiency of light-driven processes in supercapacitors. This is achieved by enabling lighter to interact with a wider volume of the electrode material, hence enhancing photothermal or photoelectrochemical reactions. Implementing this can enhance the energy storage capacity and overall performance of the supercapacitor.

##### Refractive index

**Fig. 4:**
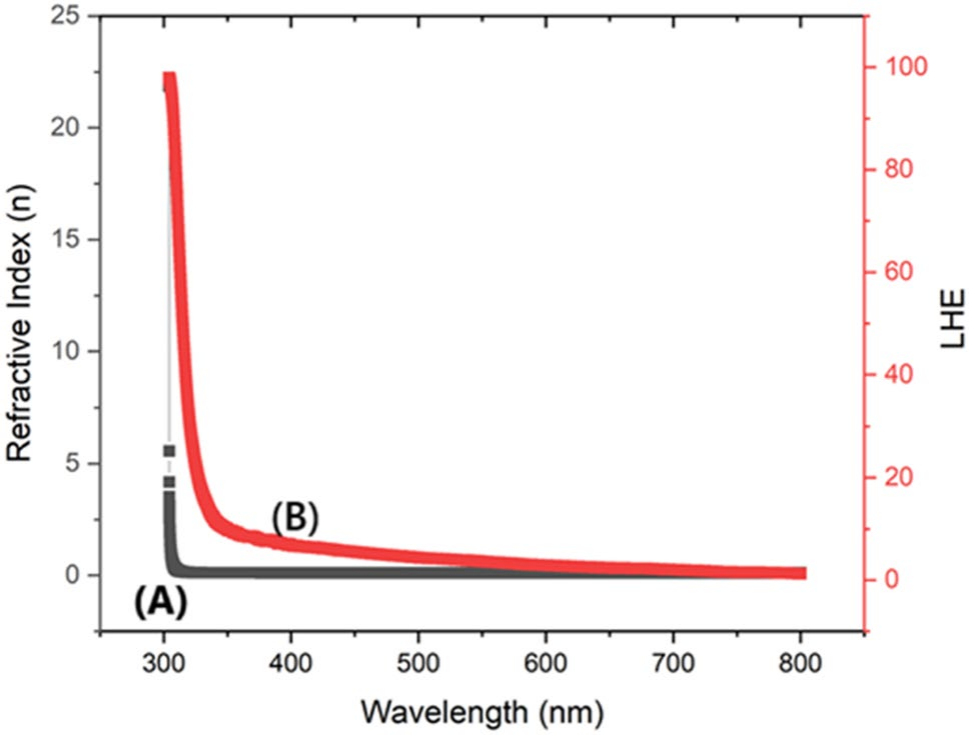
(**A**) BPEP Refractive index, (**B**) Light Harvesting energy of BPEP.

The refractive index of a substance denotes its ability to decrease the speed of light as it traverses through it, and is strongly correlated with the absorption of ultraviolet (UV) radiation which is shown in Fig. 4A. Materials with a high UV absorbance value generally exhibit a high refractive index due to the increased excitation of electrons by incoming light, resulting in a greater slowing down of the light. The refractive index reaches its peak value of 22.5 (Fig. 4A) at a wavelength of 300 nm, most likely because to the presence of intense electronic transitions or resonances at this specific wavelength. When the wavelength exceeds 300 nm, these transitions become less efficient, resulting in a significant decline in the refractive index. In the context of supercapacitor applications, having a high refractive index at specific wavelengths can boost the interaction between light and matter in the electrode materials. This, in turn, can accelerate photochemical reactions. This can lead to enhanced charge storage efficiency and overall performance of the supercapacitor.

##### LHE

Light Harvesting Efficiency (LHE) quantifies the effectiveness of a substance in absorbing and converting light energy into different forms, and it is directly related to the values of UV absorption. For BPEP the light harvesting efficiency (LHE) is plotted and shown in Fig. 4B. A greater UV absorbance generally suggests superior light absorption and hence higher light harvesting efficiency (LHE). At a wavelength of 300 nm, the LHE exhibits a peak absorption of 99 (6B), indicating ideal absorption resulting from pronounced electronic transitions or resonances. Once the wavelength surpasses 300 nm, these transitions become less efficient, resulting in a gradual reduction in LHE until it reaches a minimum. Materials having high light harvesting efficiency (LHE) at specific wavelengths can enhance the absorption of light within the electrode materials in supercapacitor applications. This, in turn, improves photochemical processes and increases the efficiency of charge storage. This feature allows for the more effective use of light energy, resulting in improved performance and energy storage capacity of the supercapacitor.

##### Optical transition strength

**Fig. 5:**
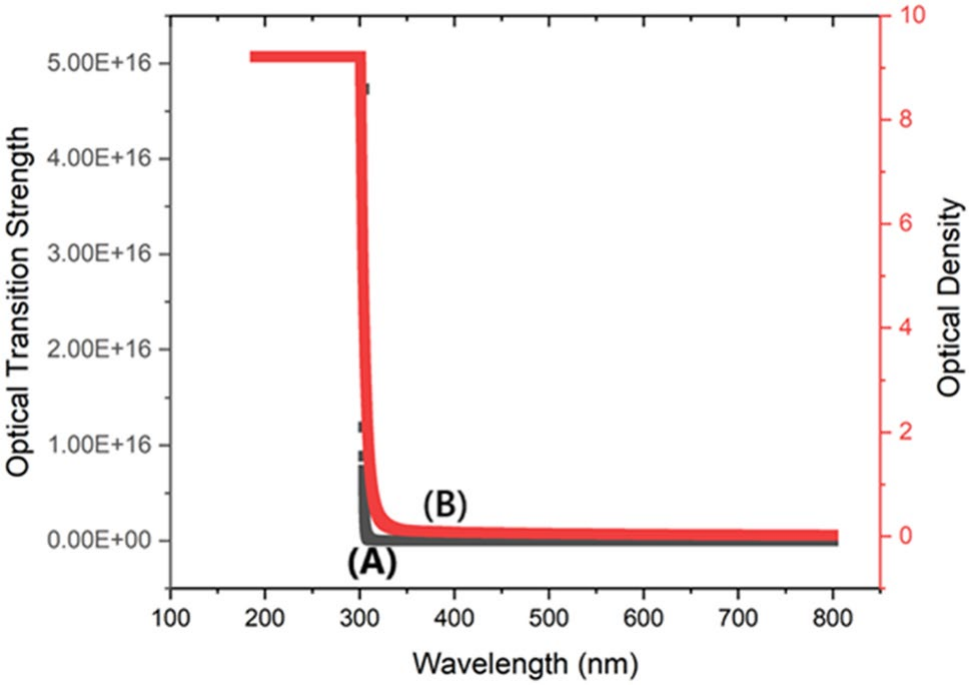
(**A**) Optical transition strength, (**B**) Light absorbance capacity.

Optical transition strength is a measure of the likelihood of electronic transitions taking place in a material upon absorption of light, and it is strongly associated with UV absorbance which is shown in figure. A high UV absorbance value often indicates a significant intensity of optical transitions, implying a strong optical transition strength. At a wavelength of 300 nm, the optical transition strength hits its peak at 800,000 (Fig. 5A), most likely because of notable electronic transitions or resonances occurring at this specific wavelength. When the wavelength exceeds 300 nm, the likelihood of these transitions decreases, resulting in a sharp decline of the optical transition strength to zero. Materials possessing a high optical transition strength at specific wavelengths might improve the effectiveness of light-induced processes, such as photothermal or photoelectrochemical reactions, in supercapacitor applications. Enhancing the efficiency of the supercapacitor can enhance its energy storage capacity and overall performance by facilitating more efficient absorption and conversion of light within the electrode materials.

##### ODU

The ODU of BPEP is shown in Fig. 5B. The Optical Density Unit (ODU) is a quantitative measure of the amount of light that is absorbed by a substance, which is directly correlated to the values of UV absorbance. A high UV absorbance corresponds to a high optical density unit (ODU), indicating that the substance efficiently obstructs light. At a wavelength of 300 nm, the ODU exhibits a peak value of 9.2 (Fig. 5B) because of notable electronic transitions or resonances, which in turn leads to the highest level of light absorption. When the wavelength exceeds 300 nm, these transitions become less efficient, resulting in a progressive drop of the ODU to zero. Materials having high optical density at specific wavelengths can increase the absorption of light in electrode materials, hence improving photothermal or photoelectrochemical reactions in supercapacitor applications. The heightened light absorption can enhance the efficacy of energy storage and overall performance of the supercapacitor by facilitating a more efficient interaction between light and the active materials.

##### SELF and VELF

**Fig. 6:**
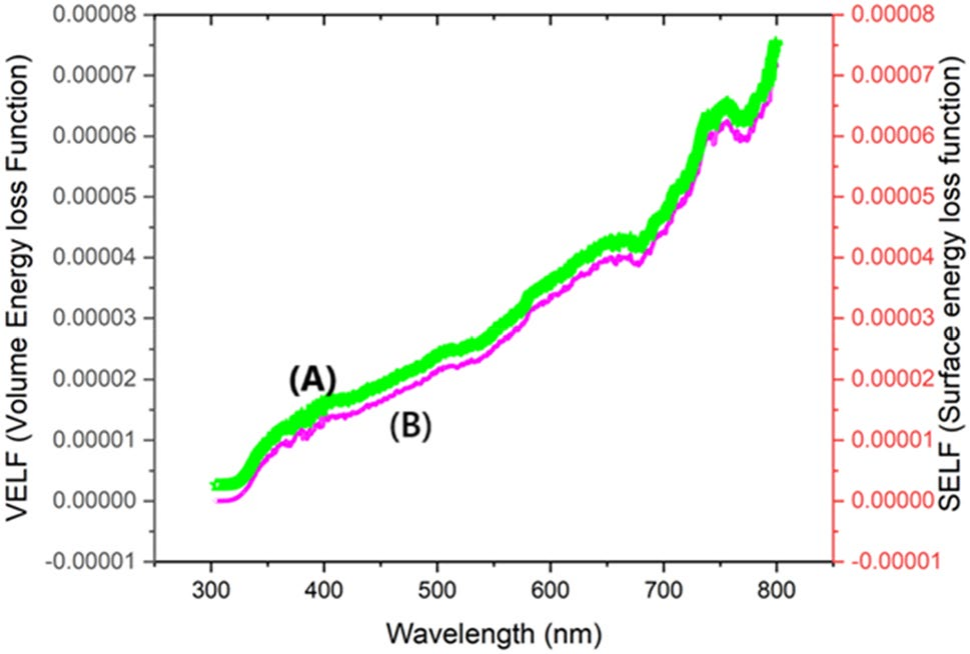
(**A**) VELF of BPEP, (**B**) SELF of BPEP.

In the given figure, SELF and VELF quantify the amount of energy that electrons lose when they contact the surface and volume of a material, respectively. These measures are directly linked to the values of UV absorbance. Greater UV absorbance often indicates higher energy dissipation resulting from stronger electrical interactions. At a wavelength of 350 nm, both SELF and VELF exhibit their highest values shown in Fig. 6A and 6B, with SELF reaching 7.1510^–5^ and VELF reaching 7.2210^–5^. This is likely attributed to significant electronic transitions occurring at this specific wavelength. As the wavelength exceeds 350 nm, the effectiveness of these transitions diminishes, resulting in a sharp decline of both SELF and VELF values to zero. Materials with high self-absorption and velocity of electron flow (SELF and VELF) at specific wavelengths can improve energy dissipation and electron interaction inside electrode materials in supercapacitor applications. This optimization of energy transfer processes enhances charge storage efficiency and overall supercapacitor performance.

The sudden drop of SELF from 0.00007 to 0.00004 suggests a rapid decline in energy dissipation due to the diminished excitation of electrons. This behavior could indicate a threshold beyond which photon energy is no longer sufficient to cause significant surface energy loss, highlighting a cutoff point in the material’s optical interaction efficiency.

#### FMO analysis

FMO analysis is important for deciding ligands’ kinetic stability & chemical susceptibility. Exploring the energies & charge transfer events from the HOMO to the LUMO is likewise critically dependent on it^33,34^. The HOMO is electron-rich and capable of donating electrons, whilst the LUMO can take electrons. Figure S2 displays the 3-D plots that resulted from the FMO, together with their band gap (Eg). The positive and negative orbital phases are depicted in these figures, respectively, by the hues red and green. Upon close inspection, Figure S4 reveals that the LUMO is positioned above the picolinic acid atoms, but the HOMO is situated on the benzimidazole ring and the transfer happens through N_15_-H_24_…O_2_ wherein responsible for the stability. BPEP’s HOMO energy is −6.202 eV. Apart from the individual energies, the HOMO–LUMO Eg affects the thermal endurance and chemical responsiveness of the compound. More stability and chemical hardness are indicated by a bigger band gap. Furthermore, greater donor–acceptor configuration strength and a broad π-conjugation are likely to contribute to a reduction in FMO orbital energy gaps.

The electron affinity (EA) & ionization potential (IP) metrics for a chemical are correlated with the low values of its LUMO & HOMO, accordingly, as defined by Koopman’s theorem. Koopmans’ theorem calculates the chemical hardness(η) & softness(S) of the compound, which are important factors for understanding reactivity^35^. There is a close relationship between the band gap & (η); a superior band gap remains related by higher hardness & therefore lower reactivity. A chemical is often more reactive when its S and η values are high. Table 5 has the values of η & S, which show that our BPEP molecule is reactive. Higher electrophilicity is exhibited by molecules having greater ω values, while nucleophilicity is indicated by molecules having lower ω values^31^. By having the greatest ω value (5.249 eV), the BPEP molecule exhibits a particularly electrophilic character. Ability of a molecule to draw electrons is measured by its electronegativity index(χ). A molecule’s chemical potential, or μ, which indicates an atom’s capacity to donate and take energy, is another way to verify kinetic stability^36^. Increased molecular potential compounds lose electrons more readily than lower molecular potential compounds. The BPEP compound is stable and difficult to break down, as indicated by the computed negative value of μ (−4.377 eV).

**Table 5:** Calculated energy values of title compound by B3LYP/6–311 + + G(d, p) for gas and solvents.

Parameters (eV)	Gas
E_HOMO_	−6.202
E_LUMO_	−2.552
Energy band gap (ΔE)	3.65
Ionization potential	6.202
Electron affinity	2.552
Chemical hardness	1.825
Chemical Softness	0.274
Electro negativity	4.377
Chemical potential	−4.377
Electrophilicity index	5.249
Electron donating capability (ω^-^)	7.665
Electron accepting capability (ω^+^)	3.28
Net electrophilicity (${\upomega }_{-}^{+}$)	10.95
Nucleophilicity index (N)	0.191

#### NBO analysis

NBO analysis was utilized to comprehend the characteristics of the donor–acceptor orbital linkages within the composites^37,38^. Second-order perturbation energy (E^(2)^) is employed to calculate the degree of a relationship among donor & acceptor orbitals^39^. Greater stabilization energies are associated with enhanced adsorption of the sensing material because they show greater interactions amongst the supplier and acceptor orbitals. Electrons delocalize from σ(N_16_-C_22_) to σ*(C_18_-C_20_), σ (C_20_-C_23_) to σ*(N_16_-C_18_), σ(C_5_-H_10_) to σ*(N_3_-C_4_), (N_15_-C_22_) to σ*(C_17_-C_19_), and σ (C_8_-H_13_) to σ*(N_3_-C_4_), resulting in E^(2)^ values of 5.63, 5.36, 5.21, 4.93, and 4.85 kcal/mol. Electron transfer from lone pairs LP^(1)^ on N_3_, N_16_, O_1_, and N_16_ to anti-bonding acceptors σ*(C_4_-C_5_), σ*(C_7_-C_8_), σ* (N_15_-C_22_), σ*(O_2_-C_9_), and σ*(C_17_-C_18_) results in (E^(2)^) stabilization energies of 9.9, 9.01, 7.83 & 7 kcal/mol, respectively, which have a major impact on the resonance in the molecule shown in Table 6. Nevertheless, the stabilization energies are comparatively large, at 30.3 and 15.88 kcal/mol, accordingly, when electrons from lone pairs LP^(2)^ on O_2_ are given to anti-bonding acceptors σ* (O_1_-C_9_) and σ*(C_4_-C_9_). The nitrogen atom lone pairs play a critical role in the BPEP molecule. There is a noticeable energy delocalization during the transitions from lone pairs LP^(1)^ on N_15_ to π* [N_16_-C_22_, C_17_-C_18_], with E^(2)^ values of 47.42 and 33.7 kcal/mol, respectively. C9 = O1 (1.9967 e) and C9-O2 (1.9956 e) both have significant double bond properties. NBO analysis revealed an excellent N_15_-H_24_‧‧‧O_2_ intermolecular H-bond caused by orbital overlap among a lone pair LP^(1)^O_2_ → σ*(N_15_-H_24_), with a stabilization energy of 5.8 kcal/mol, which improves the molecule’s reactivity. For BPEP, the transitions comprising π*-π* connections (π*(C_17_-C_18_) to π*(C_20_-C_23_), π*(N_3_-C_8_) to π* [C_4_-C_5_, C_6_-C_7_], & π*(N_16_-C_22_) to π*(C_17_-C_18_)) have the least occupancy states and the largest stabilization energies. For these gas phase transitions, the calculated occupancies & E^(2)^ were 0.22861, 0.11740, 0.37309, 0.38231, 0.36049, and 297.92, 215.27, 197.51, 83.72 kcal/mol, respectively. These interactions improve the molecule’s structural stability, maintaining a charge transfer connection^40,41^.

**Table 6:** Second order perturbation theory analysis of Fock matrix in NBO basis.

Type	Donar (i)	ED (i) (e)	Type	Acceptor (j)	ED (j) (e)	E (2)^a^ kcal/mol
π*	C_17_-C_18_	0.47450	π*	C_20_-C_23_	0.30834	297.92
π*	N_3_-C_8_	0.34570	π*	C_4_-C_5_	0.33418	215.27
π*	N_3_-C_8_	0.34570	π*	C_6_-C_7_	0.29085	197.51
π*	N_16_-C_22_	0.32727	π*	C_17_-C_18_	0.47450	83.72
LP^(1)^	N_15_	1.60276	π*	N_16_-C_22_	0.32727	47.42
LP^(1)^	N_15_	1.60276	π*	C_17_-C_18_	0.47450	33.7
LP^(2)^	O_2_	1.86555	σ*	O_1_-C_9_	0.08170	30.3
π	C_6_-C_7_	1.63040	π*	N_3_-C_8_	0.34570	26.03
π	N_3_-C_8_	1.71231	π*	C_4_-C_5_	0.33418	24.94
π	C_6_-C_7_	1.63040	π*	C_4_-C_5_	0.33418	20.08
π	C_20_-C_23_	1.72109	π*	C_19_-C_21_	0.33500	19.96
π	C_4_-C_5_	1.61957	π*	C_6_-C_7_	0.29085	19.41
π	C_19_-C_21_	1.73437	π*	C_17_-C_18_	0.47450	19.16
π	C_17_-C_18_	1.58878	π*	C_19_-C_21_	0.33500	18.9
π	C_4_-C_5_	1.61957	π*	N_3_-C_8_	0.34570	18.85
π	C_17_-C_18_	1.58878	π*	C_20_-C_23_	0.30834	18.63
π	N_16_-C_22_	1.98476	π*	C_17_-C_18_	0.47450	18.23
π	C_20_-C_23_	1.72109	π*	C_17_-C_18_	0.47450	18
π	C_19_-C_21_	1.73437	π*	C_20_-C_23_	0.30834	17.12
LP^(2)^	O_2_	1.86555	σ*	C_4_-C_9_	0.07544	15.88
π	C_17_-C_18_	1.58878	π*	N_16_-C_22_	0.32727	14.58
π	N_3_-C_8_	1.71231	π*	C_6_-C_7_	0.29085	14.13
LP^(1)^	N_3_	1.91368	σ*	C_4_-C_5_	0.03075	9.9
LP^(1)^	N_3_	1.91368	σ*	C_7_-C_8_	0.02585	9.01
LP^(1)^	N_16_	1.92578	σ*	N_15_-C_22_	0.03351	7.83
LP^(1)^	O_1_	1.97591	σ*	O_2_-C_9_	0.02014	7
σ	C_19_-C_21_	1.97555	σ*	N_15_-C_17_	0.02596	6.55
LP^(1)^	N_16_	1.92578	σ*	C_17_-C_18_	0.03799	5.87
LP^(1)^	O_2_	1.97035	σ*	N_15_-H_24_	0.03036	5.8
σ	N_16_-C_22_	1.98476	σ*	C_18_-C_20_	0.02298	5.63
σ	C_20_-C_23_	1.97697	σ*	N_16_-C_18_	0.02087	5.36
σ	C_5_-H_10_	1.97815	σ*	N_3_-C_4_	0.02275	5.21
σ	N_16_-C_18_	1.97727	σ*	C_22_-H_27_	0.02152	5.11
σ	N_15_-C_22_	1.98907	σ*	C_17_-C_19_	0.02152	4.93
σ	C_8_-H_13_	1.98189	σ*	N_3_-C_4_	0.02275	4.85
σ	C_17_-C_18_	1.96577	σ*	C_17_-C_19_	0.02152	4.62

^a^E^(2)^ means energy of hyper conjugative interaction (stabilization energy).

#### Electron–hole analysis

Discovering how electrons and holes behave can help us understand the properties of electron excitation^42^. Multiwfn^17^, a program that emphasizes intense excitation inside the molecules themselves, is used to assess the charge transfer characteristics of BPEP. In Fig. S5, isosurface diagrams depict the electron–hole distributions in the initial four excited states of the gas phase, and relevant statistics from these simulations are presented in Table 7. According to Figure S5, in the first, second, & fourth excited states, holes are predominantly contained to the benzimidazole ring atoms, whereas electrons accumulate among the picolinic acid atoms. In the third excited state, electrons and holes are limited to the picolinic acid ring atoms. The D-index, which gauges the electron–hole transmission length, is a crucial statistic in this investigation. The maximum D-index value for BPEP occurs in the fourth excited state. Increased values of the Δr index, another crucial characteristic, demonstrate the qualitative electron excitation mode; more CT throughout the molecule for this state is apparent by increased Δr values. The fourth excited state is where the excitation energy and Δr index peak for BPEP, suggesting that this state has the highest excitation through transitions.

**Table 7:** Excitation energy (E), D index, Dr index, t index, Sr function for different excited states for the title compound in gas phase.

Excited state	Excitation energy E (eV)	Charge transfer length D index (A°)	Δr index (A°)	t index (A°)	Sr (a.u)	Sm (a.u)	H index
I	3.183	5.968	11.312	4.847	0.033	0.003	2.198
II	3.278	5.932	11.305	4.754	0.038	0.004	2.143
III	4.072	0.994	1.974	−0.193	0.504	0.244	2.001
IV	4.134	6.451	12.167	5.217	0.042	0.003	2.069

### Photoluminescence

The photoluminescence spectrum of BPEP is shown in a figure which has three clearly distinguishable peaks at wavelengths of 396.23 nm, 418.24 nm, and 647.99 nm. These peaks have corresponding intensities of 10.97, 11.10, and 314.14, (Figure S6) respectively. The peak at 647.99 nm is the most noticeable and indicates a powerful emission in the red part of the spectrum, suggesting important electronic transitions occurring within the material. The comparatively greater intensity at this particular wavelength in comparison to the others indicates a higher likelihood of radiative recombination processes. The luminescence properties of a material can have significance for supercapacitors, as they may be connected to the electrical structure and charge transfer capacities of the material. Efficient electron mobility and stability, crucial for high-performance supercapacitors, can be indicated by enhanced photoluminescence. The material’s capacity to undergo such changes indicates its potential for high energy density and improved charge–discharge cycles, hence enhancing the overall efficiency and longevity of supercapacitors.

### Cyclic voltametric analysis

**Fig. 7:**
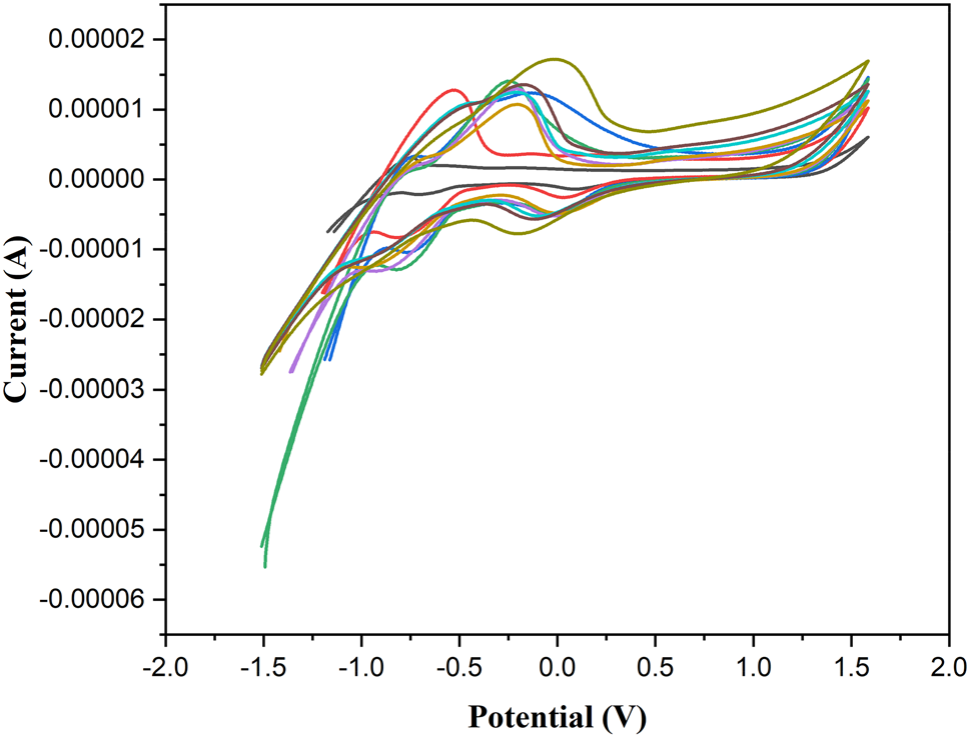
Cyclic volumetric analysis of BPEP.

The cyclic voltammogram was captured to assess the electrochemical behavior of the fabricated substances. The CV of the newly synthesized electrode materials is depicted in Fig. 7, which was obtained at varying scan rates within a potential range of 0–0.66 V in 1 M aqueous KOH. The potential window of capacitance behavior is a crucial element in the advancement of supercapacitance. The curves exhibited a striking similarity in their shapes. With an increase in the scan rate from 10 to 200 mVs^−1^ shown in Fig. 7, a balanced redox peak was noted, signifying the commendable rate performance of all the fabricated electrode materials. In a basic electrolyte solution, an enhanced ion diffusion rate does indeed aid in promoting favorable Faradic redox reactions. This is further confirmed by the observed displacement of the redox peaks. The specific capacitance values, measured in Fg^−1^, at various scan rates are as follows: 125.45 at 10 mV/S^−1^, 118.96 at 20 mV/S^−1^, 113.64 at 30 mV/S-1, 87.60 at 40 mV/S^−1^, 61.23 at 50 mV/S^−1^, 49.73 at 60 mV/S^−1^, 44.196 at 80 mV/S^−1^, 38.425 at 100 mV/S^−1^, and 25.689 at 200 mV/S^−1^. The electrochemical performance of BPEP appears promising due to the interactions between the following bonds, like N_15_-H_24_‧‧‧O_2_ among the others. These values were determined respectively and then it is compared with the existing material shown in Table 8. From this we can confirm that the synthesized BPEP exhibits higher capacitance value which is shown in Table 8^43–46^. In the fabricated materials, the calculated Cyclic Voltammetry (CV) displayed nearly identical redox peaks, suggesting that the capacitance is influenced by pseudocapacitive behavior. The scan rate can indeed influence the specific capacitance value. This, in turn, can enhance the electrical conductivity of the entire electrode and the rate of electron transport, contributing to its pseudocapacitive nature. This pseudocapacitive nature is further confirmed by the presence of oxidation and reduction peak presence which is appeared to be at −0.522 and 0.02 respectively. The donation and acceptance of the electrons explain the synthesized BPEP is applicable for energy storage materials. While high surface area and porosity are key for rapid charge–discharge cycles, excessive porosity can lead to mechanical fragility and lower energy density. Future studies could provide further insights into the optimal porosity range for maximizing BPEP performance.

**Table 8:** Comparison of specific capacitance with existing material.

Material	Specific capacitance	Ref
Benzimidazole-Based Polyimide Membrane	121 F/g	Ref. 46
BI-rGO electrode	252 µF/g	Ref. 47
N dopped graphene oxide and 3,3′,4,4′-tetraaminodiphenyl oxide (TADPO)	340 F g^−1^	Ref. 48
N-doped graphene by benzimidazole	256 F g − 1	Ref. 49

### Nyquist plot

**Fig. 8:**
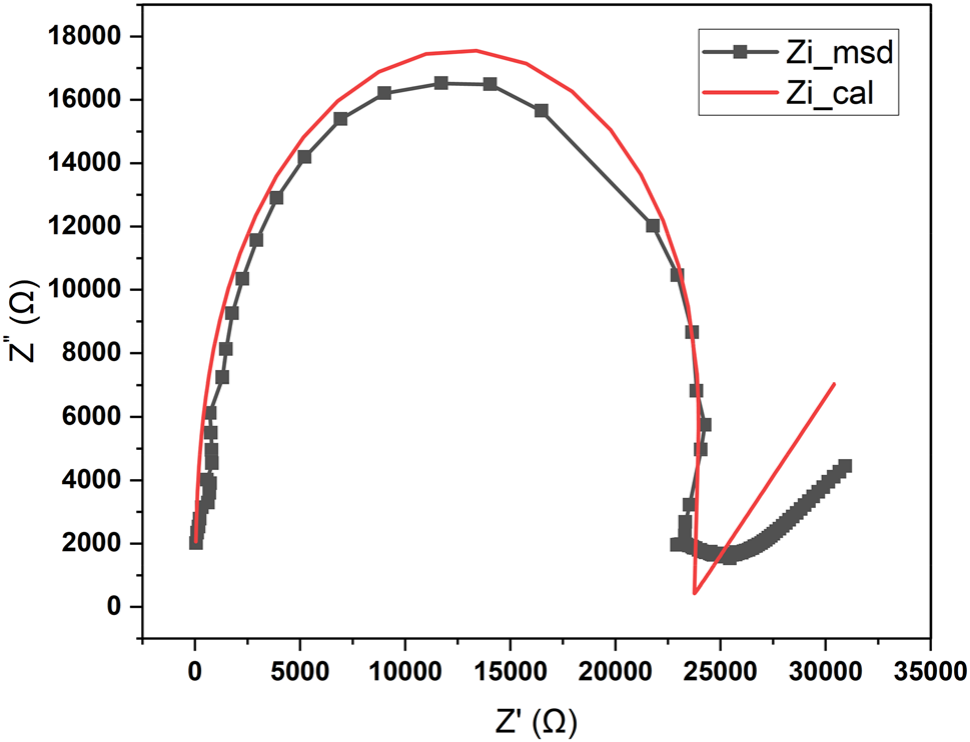
Nyquist Plot of BPEP.

The electrochemical system is analyzed by Electrochemical Impedance Spectroscopy (EIS) by applying a minor voltage perturbation. This approach elucidates the electron transfer processes in the produced material across a frequency spectrum of 1 MHz to 0.1 Hz. The Nyquist plot for BPEP is illustrated in the figure. The semicircle in the plot verifies the existence of bulk and grain limits. The semicircle at elevated frequencies represents the charge transfer resistance, whereas the tail at reduced frequencies is ascribed to ion diffusion. The plot also illustrates redox reactions, ion interactions, and ion transport at the interface between BPEP and the electrolyte. The augmented space between data points at elevated frequencies is attributable to the chosen frequency range and equipment constraints during high-frequency impedance measurements, leading to a sparser data distribution in that area. The observed sudden decrease in values is ascribed to prevailing inductive behavior, as seen in the analogous circuit model. The Nyquist plot of the synthesized BPEP was modeled using the Randles equivalent circuit, as illustrated in Fig. 8. This circuit comprises two resistors, one inductor, and one capacitor configured in three parallel branches, which are connected in series with a Warburg impedance. In the initial branch, the resistor and inductor are arranged in parallel. The second branch has a resistor that is additionally connected in parallel with the first branch. The third branch has a capacitor, which is also in parallel with the preceding branches. The behavior of this analogous circuit is defined by a semicircle at high frequencies and an arch at low frequencies. This shift from capacitive to inductive response is a characteristic phenomenon in electrochemical systems.

The dielectric constant variation with respect to log f is shown in figure in S5 and formula^1^.

1 $${\epsilon }_{r}=\frac{cd}{A{\epsilon }_{o}}$$

where c represents capacitance in Farads, d denotes the material thickness in meters, and ε0 signifies the permittivity of free space. A- Area of the sample in m^2^. Based on the graph, it is evident that the dielectric constant reduces as the frequency increases. High dielectric constant ascribed to the presence of all four types of polarisations: space charge, orientation, electronic, and ionic polarisations.

### Interaction analysis

#### NCI- RDG analysis

The graphical determination of Non-Covalent Interactions (NCI) among various entities in a compound is analyzed using Reduced Density Gradient (RDG) analysis. This method provides insights into weak NCIs in real space and delineates areas where different interactions occur. By employing straightforward color codes, RDG analysis differentiates among hydrogen bonding, vdW also repulsive steric interactions^20^. The investigation makes use of VMD 1.9.4 software for visualization and cube files created by the Multiwfn 3.8 program for analysis. To study weak interactions in physical space, Johnson and colleagues used RDG analysis. Given relation^47^.

$$\text{RDG }(\text{r})=\frac{1}{{2(3\pi {r}^{2})}^\frac{1}{3}}\frac{|\nabla {\ell}|}{{(r)}^\frac{3}{4}}$$

This compound’s RDG isosurfaces also NCI scatter plots are shown in Figure S4. Strong repulsion is represented by the red areas, strong attraction by the blue areas, and electrostatic interaction by the green areas. The product of the sign of λ2 also ρ results in Sign(λ2)ρ. From the figure S6, we observe that the sign(λ2)ρ values range from −0.05 to 0.05 a.u. A negative sign(λ2)ρ, which indicates attractive interactions such as hydrogen bonds, is represented by the left side blue spikes, with a notable negative value of −0.03 a.u. Conversely, the right side red spikes, indicating repulsive interactions, correspond to a positive sign(λ2)ρ with the smallest positive value of 0.016 a.u. Strong and advantageous interactions, especially in the N–H…O hydrogen bonds, are indicated by red patches. vdW interactions between the carboxylic acid group are indicated by green colors on the RDG isosurface, which range from − 0.01 to 0.01 a.u. The high stability of BPEP is indicated by a combination of strong hydrogen bonding, vdW, and steric interactions. The RDG isosurface map reveals an elliptical red spot near the center of the ring, which is attributed to destabilizing steric interactions. Green elliptical regions suggest occurrence of weak vdW interactions among hydrogen atoms^48,49^.

#### ELF and LOL analysis

Surface topological analysis is manifested using ELF and LOL Analysis. Using Multiwfn software package, ELF and LOL have a high probability of revealing an electron pair on molecular surface. The portrayed colour map is shown in the given figure S7. ELF and LOL share the same chemical function because they both are based on the concept of kinetic energy density. The probability density of electron pairs are measured using ELF, which highlights the area where the electron pairs are likely to be found^50^. On the other hand, by analysing the gradient of these orbitals LOF identifies the maximum overlap between localized orbitals. The ELF colour map is deliberated from 0.0 to 1.0 range; and also, regions with values below 0.5 indicate delocalized electronic regions. Conversely, the LOL reaches high values greater than 0.5 in areas where the electron density is primarily characterized by electron localization. Observing the color shades of ELF and LOL maps reveals the presence of bonding and nonbonding electrons. Specifically, the red color surrounding hydrogen atoms indicates the presence of these electrons. The red color cloud around the hydrogen atoms indicates the high ELF and LOL values, suggest a significant concentration of electrons in that particular area^51^. The red color around hydrogen atoms in Figures S7 indicates the existence of both bonding and nonbonded electrons. On the other hand, the low electron localization value is indicated by the blue color cloud around the nitrogen atoms and the carbocation. The electron density has surpassed the color scale limit, as indicated by the white area surrounding the center of the N_15_ atoms in Figure S7. Strong electron localization resulting from covalent bonds and lone pairs of electrons is indicated by a red zone on an Electron Localization Function (ELF) map surrounding hydrogen atoms (H_12_, H_11_, H_10_, H_26_, H_26_, H_29_, and H_28_). Structural examinations support significant intermolecular interactions, such as the hydrogen bonds N_15_-H_24_‧‧‧O_2_ which are depicted in Figure S7 as bonding attraction points at N_15_ as they approach the ELF voids of H_24_. Similar to this, the Localized Orbital Locator (LOL) image around N_15_ shows regions of electron depletion, whereas the red zone surrounding hydrogen atoms (H_11_ and H_26_) suggests the establishment of covalent bonds.

### Conclusion

This study created BPEP and used impedance analysis to extensively assess its electrochemical properties. It was an innovative research endeavor. Due to hyperconjugative contacts, an NBO evaluation indicated considerable donor–acceptor interactions with stabilization energies of 47.42 and 33.7 kcal/mol and transitions from lone pairs LP^(1)^ on N_15_ to π* [N_16_-C_22_, C_17_-C_18_]. Positive results were obtained from these interactions for a number of topological studies, including ELF, LOL and RDG. which have shown considerable variations in the N–H…O interactions. These variations are especially noticeable in research emphasizing how important they are to impedance and cyclic voltammetry analyses. At 125.45 F/g, the specific capacitance values show promising promise in the data. Enhancing the charge storage capacity of the supercapacitor system required mastering the optimization of scan rates at 0.10 V/s. Additionally, the material’s viability for usage in supercapacitors was shown by broadband dielectric spectroscopy, which also highlighted the material’s stability, high-frequency performance, and safety properties. But like a piece of art, this creation also revealed aspects that needed refinement, such frequency response and stability. By taking care of these issues, its overall performance in different energy storage applications would be improved.

## Supplementary Information

Supplementary Information.

## Data Availability

The research data related to this paper are available with the corresponding author and will be provided upon reasonable request.
