# Supplementary material for: Structural and electrochemical investigation of benzimidazole picolinic acid derivatives for sustainable energy storage applications
Source: Sci Rep. 2025 Jul 1;15:20483. doi: 10.1038/s41598-025-01503-3 (PMC12217295; doi:10.1038/s41598-025-01503-3)
Supplement: Supplementary file 1 — Supplementary Information. [file 41598_2025_1503_MOESM1_ESM.docx]

Supplementary material

Figures


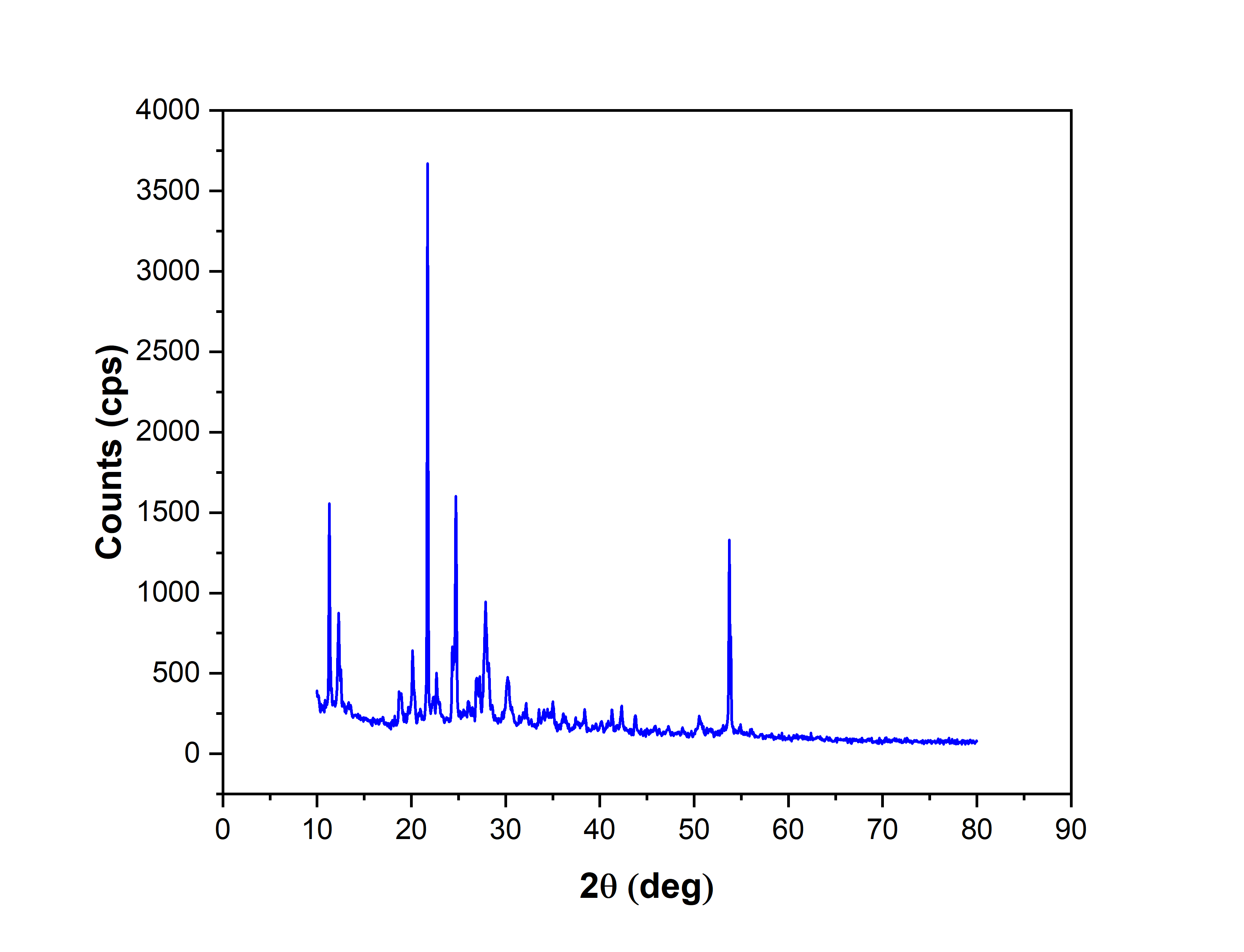


**Figure S1 Powder X-ray diffraction of** **BPEP**


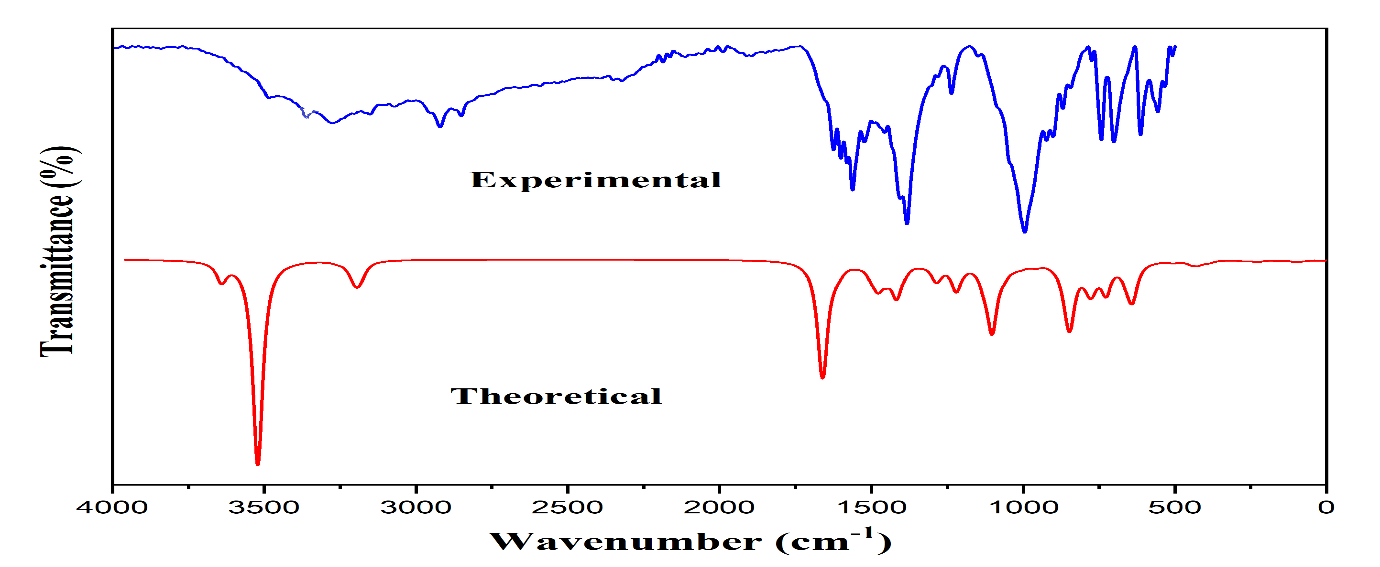


**Figure S2 Experimental and theoretical FT-IR spectrum of BPEP**


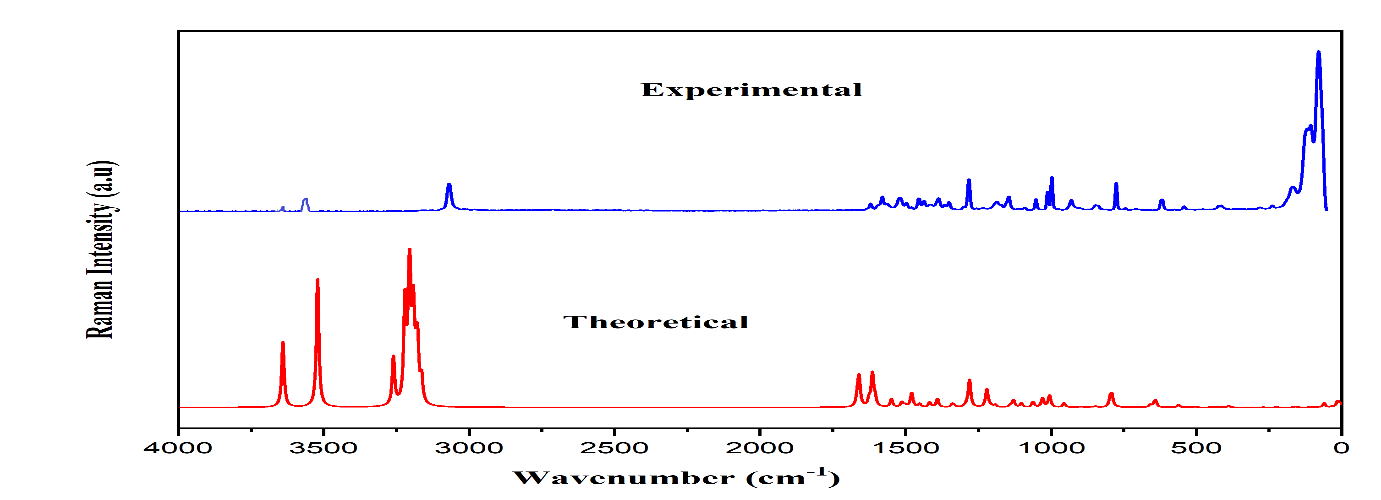


**Figure S3 Experimental and theoretical FT-Rman spectrum of BPEP**


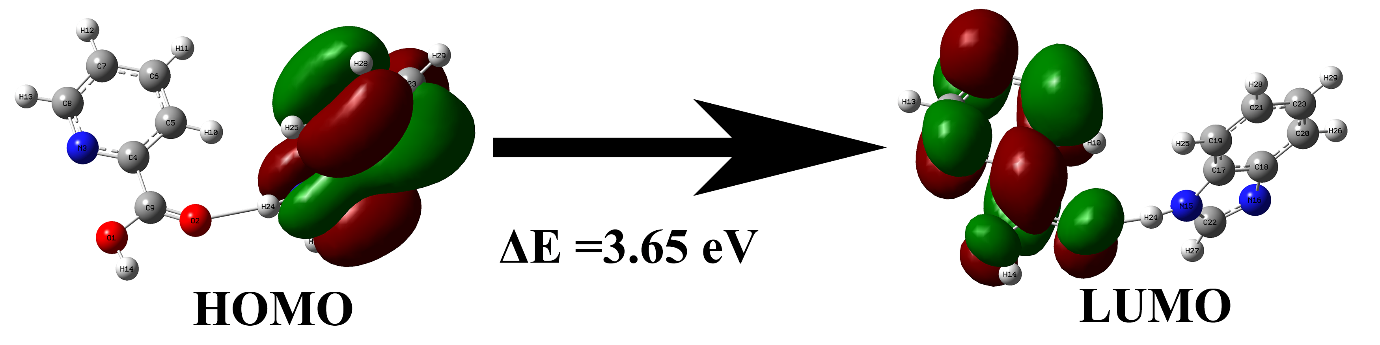


**Figure S4 HOMO-LUMO transition of BPEP**


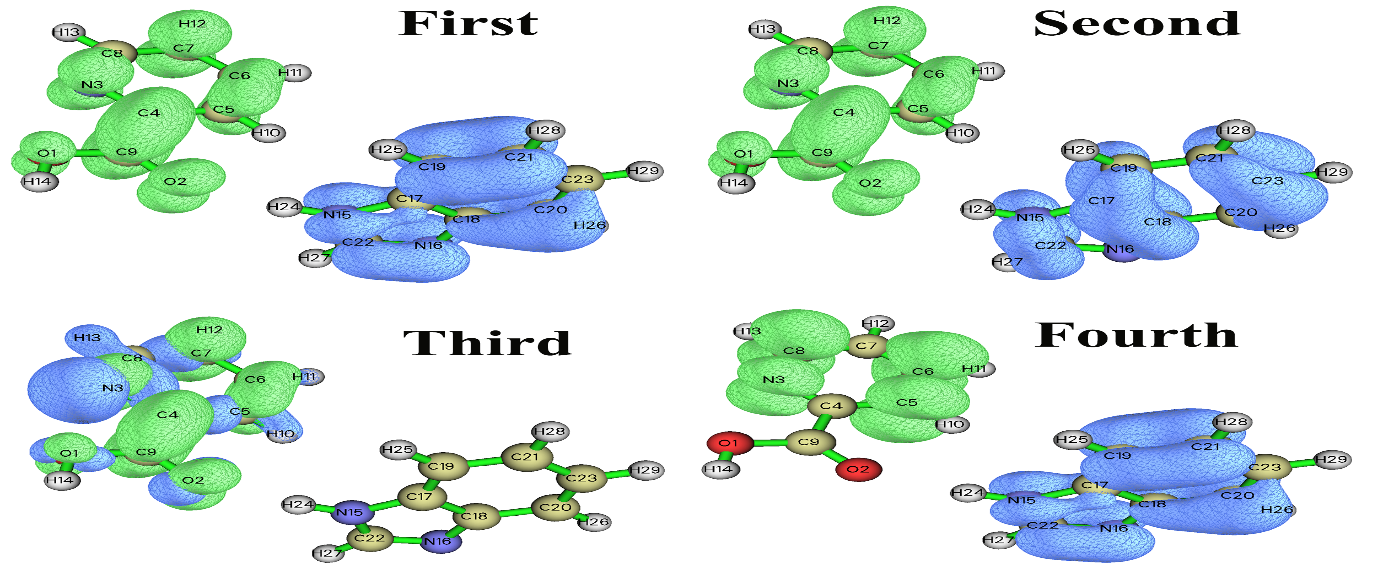


**Figure S5 Excited states of BPEP**


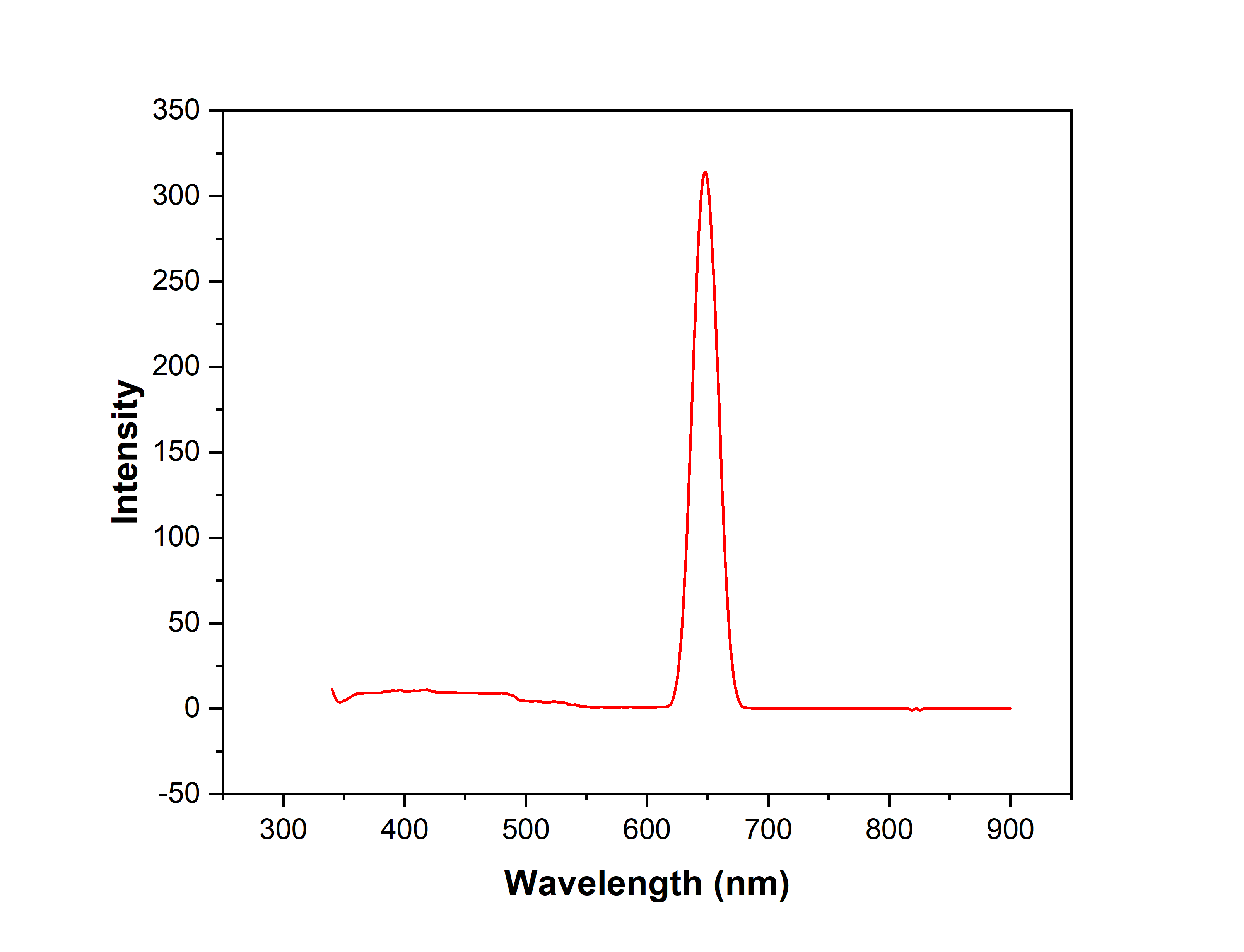


**Figure S6 Photoluminescence of BPEP**


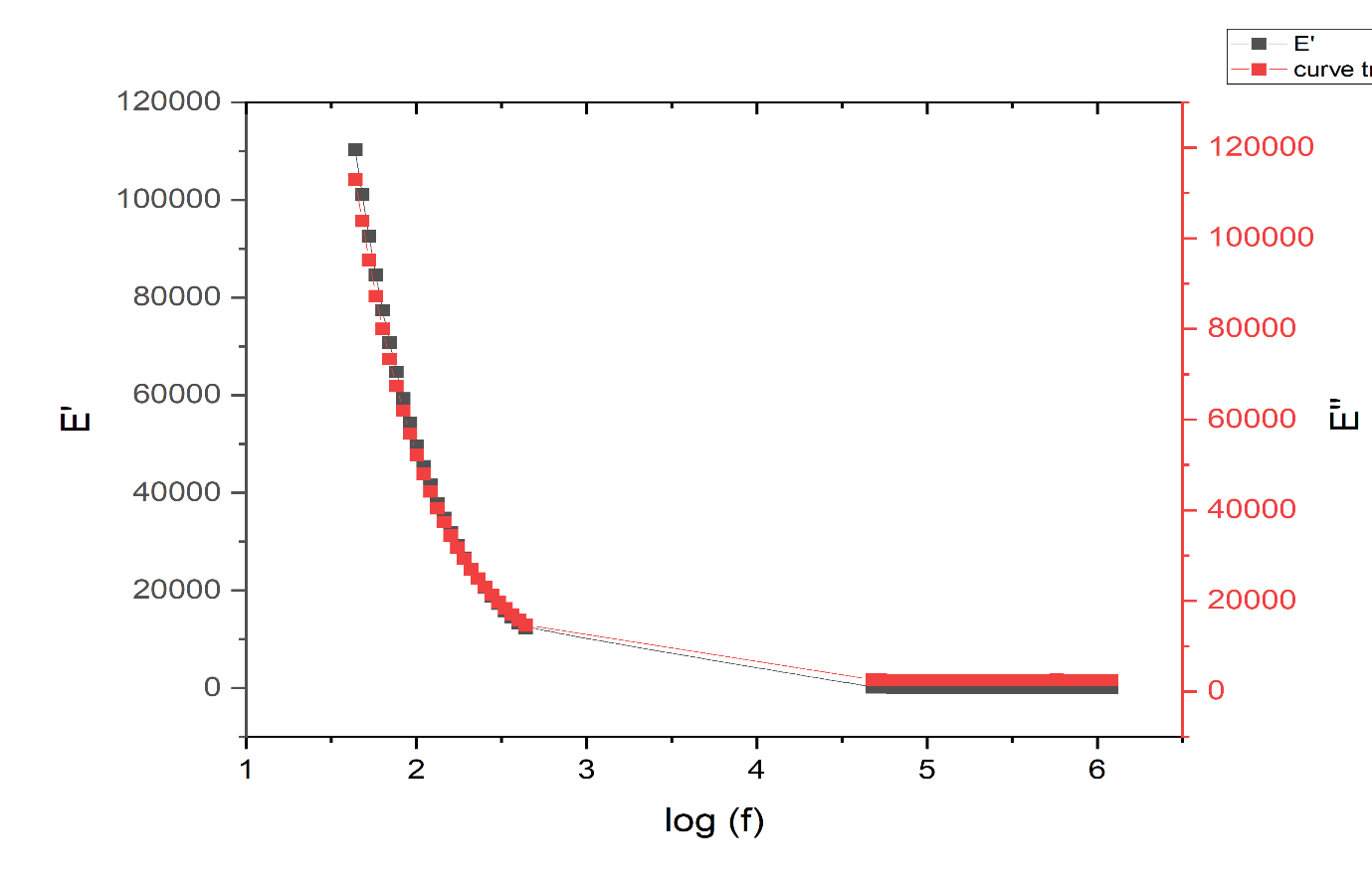


**Figure S5 Dielectric studies of BPEP**


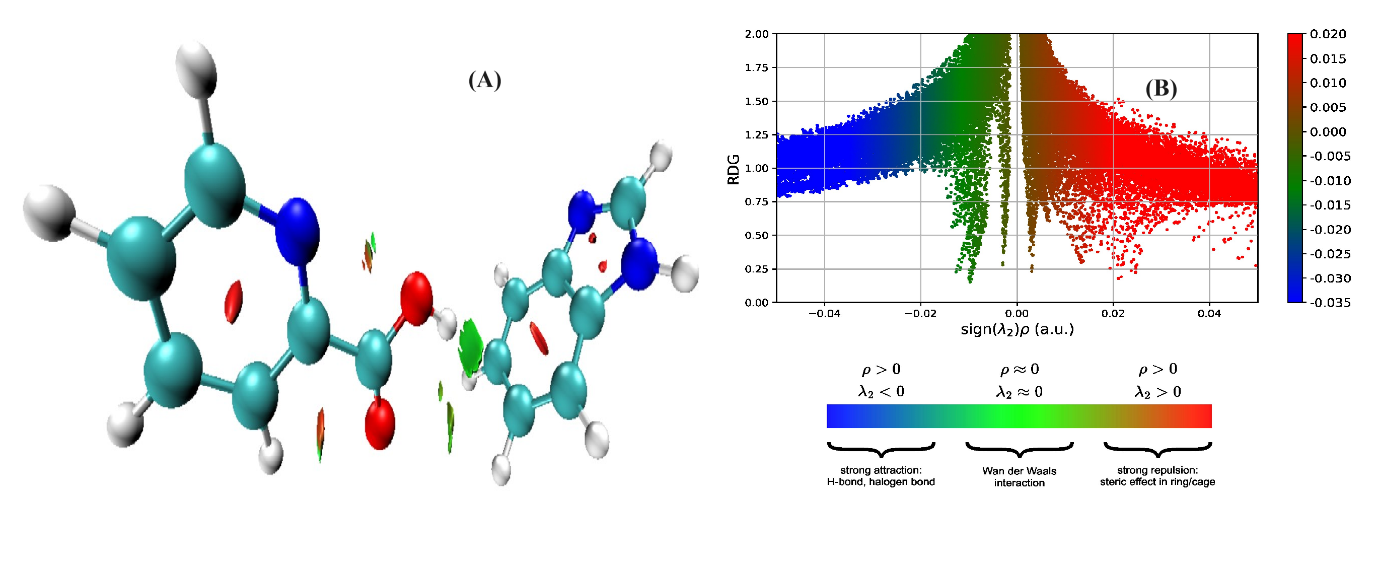


**Figure S6 2D scatter and isosurface density plots for RDG illustrating the intermolecular interactions of BPEP**


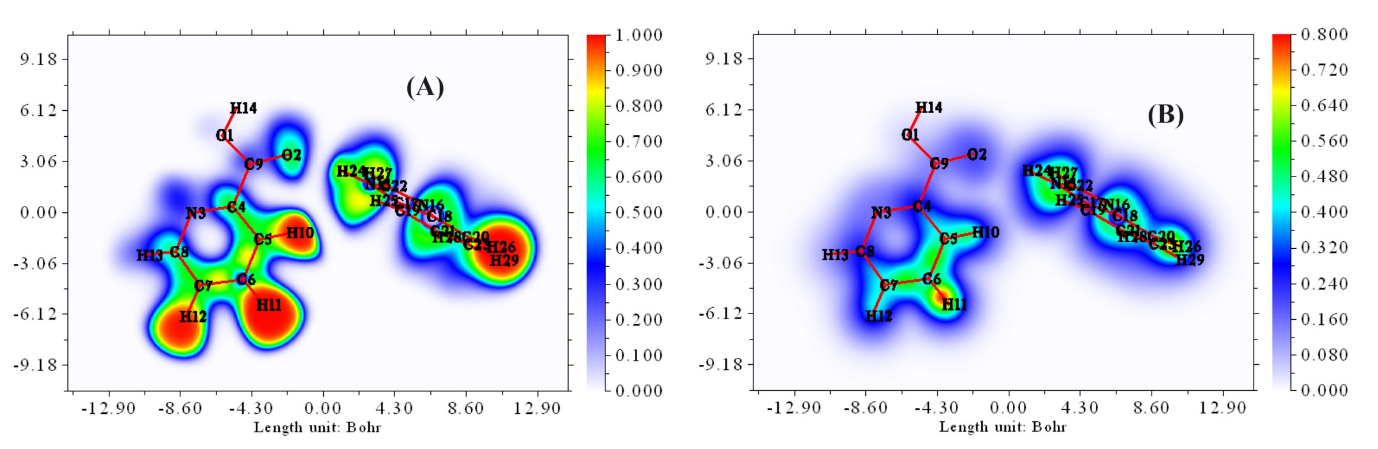


**Figure S7 Colour-filled map of ELF and LOL of BPEP**
